# Molecular Pathology of ALS: What We Currently Know and What Important Information Is Still Missing

**DOI:** 10.3390/diagnostics11081365

**Published:** 2021-07-29

**Authors:** Nikol Jankovska, Radoslav Matej

**Affiliations:** 1 Department of Pathology and Molecular Medicine, Third Faculty of Medicine, Charles University, Thomayer University Hospital, 140 00 Prague, Czech Republic; radoslav.matej@ftn.cz; 2 Department of Pathology, First Faculty of Medicine, Charles University, General University Hospital, 128 00 Prague, Czech Republic; 3 Department of Pathology, Third Faculty of Medicine, Charles University, University Hospital Kralovske Vinohrady, 100 00 Prague, Czech Republic

**Keywords:** amyotrophic lateral sclerosis, frontotemporal lobar degeneration, sporadic ALS, familial ALS, amyotrophic lateral sclerosis-frontotemporal spectrum disorder, ALS-FTSD, motor neuron disease

## Abstract

Despite an early understanding of amyotrophic lateral sclerosis (ALS) as a disease affecting the motor system, including motoneurons in the motor cortex, brainstem, and spinal cord, today, many cases involving dementia and behavioral disorders are reported. Therefore, we currently divide ALS not only based on genetic predisposition into the most common sporadic variant (90% of cases) and the familial variant (10%), but also based on cognitive and/or behavioral symptoms, with five specific subgroups of clinical manifestation—ALS with cognitive impairment, ALS with behavioral impairment, ALS with combined cognitive and behavioral impairment, the fully developed behavioral variant of frontotemporal dementia in combination with ALS, and comorbid ALS and Alzheimer’s disease (AD). Generally, these cases are referred to as amyotrophic lateral sclerosis-frontotemporal spectrum disorder (ALS-FTSD). Clinical behaviors and the presence of the same pathognomonic deposits suggest that FTLD and ALS could be a continuum of one entity. This review was designed primarily to compare neuropathological findings in different types of ALS relative to their characteristic locations as well as the immunoreactivity of the inclusions, and thus, foster a better understanding of the immunoreactivity, distribution, and morphology of the pathological deposits in relation to genetic mutations, which can be useful in specifying the final diagnosis.

## 1. Introduction

Amyotrophic lateral sclerosis (ALS) has classically been considered a disease exclusively affecting the motor system and, thus, it is a part of a group of disorders known as motor neuron diseases (MND), which includes a whole spectrum of disorders affecting upper motor neurons (corticospinal tract), lower motor neurons (in anterior horn or motor cranial nerve nuclei in the brain stem), or both [1]. The MNDs also includes “restricted” phenotypes, including primary lateral sclerosis (PLS), progressive muscular atrophy (PMA), and progressive bulbar palsy (PBP) [2]. PLS is characterized by isolated progressive upper motor neuron dysfunction without lower motor neuron symptomatology that begins in the fifth to sixth decade with progressively developing spasticity, hyperreflexia, and mild weakness [3]. Unlike ALS, PLS is most often slowly progressive (survival times of 7.2–14.5 years) [3]. PMA includes lower motor neuron dysfunction; however, upper motor neuron signs may sometimes be present [4]. Even in PMA, disease survival times are longer than in ALS [5]. PBP can be divided into childhood- and adult-onset forms [6]. The childhood-onset form, also called Fazio-Londe disease, is an autosomal recessive disease associated with progressive impairment of the cranial nerves with two clinical subtypes—an early course, typically starting before the age of 6 years with mainly respiratory symptoms, and a late course starting between 6 and 20 years with mainly motor symptoms in the upper limbs [7]. The adult-onset form attacks the bulbar region and clinically presents with swallowing, speaking, and chewing difficulties [8].

Another MND disease is spinal muscular atrophy (SMA), an inherited condition with an autosomal-recessive pattern affecting lower motor neurons that degenerate due to a lack of survival motor neuron (SMN) protein encoded by the *SMN1* gene [9]. Historically, five types of SMA, based on the age of symptoms onset and the highest physical ability achieved, are distinguished [10]. Disease severity can be modified by the *SMN2* gene, which led to the creation of the first treatment [11].

Kennedy’s disease, also known as spinal and bulbar muscular atrophy, is a recessive X-linked disease affecting men (women can be carriers). It is caused by an expansion of a CAG repeat in the gene for androgen receptors [12]. Clinically, patients present with muscle weakness, proximal atrophy in the limbs, and bulbar symptomatology. Gynecomastia due to the insensitivity to androgen, heart rhythm, and urinary problems may also be present [13].

The last condition that falls under MND is post-polio syndrome (PPS), characterized by muscle weakness and/or muscle fatigability, dysphagia, dysphonia, and subsequent respiratory failure that can begin dozens of years after recovery from a poliomyelitis infection [14].

The first to describe and diagnose ALS was Jean-Martin Charcot, so ALS is sometimes referred to as Charcot’s disease [15]. Charcot found and reported damage within the lateral columns of the spinal cord that leads to chronic progressive paralysis along with contractures but without muscle atrophy. On the other hand, lesions affecting the anterior horns cause paralysis and muscle atrophy but lack contractures [16]. Another common name for ALS is Lou Gehrig’s disease in memory of the famous New York Yankees baseball player who died (1941) of ALS at the age of 37 [17].

In ALS, the word “amyotrophy” refers to muscle fiber atrophy, caused by selective neuronal degeneration in the anterior horns of the spinal cord, leading to weakness and fasciculations of affected muscles. The phrase “lateral sclerosis” refers to the hardening of the anterior and lateral corticospinal tracts as axons and myelin are replaced by glial cells [18]. Therefore, ALS is still often defined as a fatal neurodegenerative disease characterized by progressive muscle paralysis determined by the degeneration of motoneurons in the motor cortex, brainstem, and spinal cord [19] with the characteristic limb onset form accompanied by awkwardness, weakness, and tripping or stumbling. The bulbar onset form first appears as speech or swallowing difficulties [20]. However, today, we know that not all forms are characterized solely by motor neuron lesions—some patients also have a cognitive and behavioral impairment that is remarkably similar to the behavioral variant of frontotemporal dementia (bvFTD). Recently, these cases have become referred to as amyotrophic lateral sclerosis-frontotemporal spectrum disorder (ALS-FTSD) [21]. ALS is the most common MND in adults, with the vast majority of ALS cases being sporadic; however, about 10% have a familial history with a typical Mendelian autosomal dominant pattern of inheritance [22], although cases with autosomal recessive or X-linked inheritance are also known [23]. Regarding genetic variants, the most common cause is a hexa-nucleotide repeat expansion of GGGGCC in the first intron of chromosome 9 open reading frame 72 (*C9orf72*), which causes 30–50% of familial ALS (FALS) cases and 5% of sporadic ALS (SALS) [24,25]. These repeat expansions are also frequently found in frontotemporal dementia (FTD), which shows the molecular overlap between these two pathological units, i.e., ALS and FTD [26]. Clinically, the sporadic and familial forms are indistinguishable [27], although some studies suggest that the familial variant has an earlier onset [28].

The aim of the review is to compare the neuropathological findings in individual types of ALS in detail, including characteristic inclusions, their immunoprofiles and characteristic localizations, and thus, foster a better understanding of their relationship to genetic mutations, which can be useful in specifying the final diagnosis.

## 2. Incidence and Prevalence of ALS

The incidence of ALS in Europe is reported to be around 1–2.6 cases per 100,000 inhabitants per year, and the prevalence is 6 per 100,000 inhabitants per 100,000 population [22], although foci of higher frequencies occur in the Western Pacific [29]. The disease typically occurs at 58–60 years [22], rarely occurs before the age of 40, and generally, is slightly more common in men (the reported M:F ratio is about 1.5:1) [29]; however, most studies suggest that the bulbar onset form is more common in women [30,31]. The median survival time is 20–48 months, though 10–20% of patients survive more than ten years [20].

## 3. Etiology

The cause of ALS is not yet fully understood; however, the various genes involved in the pathogenesis of ALS share similar biological functions, which allowed the identification of some major molecular pathways that trigger selective damage in specific neurons [27]. The effect of head injury [32], viruses like herpes simplex [33], enterovirus [34], or retrovirus [35], exotoxins including excitotoxicity mediated by glutamate [36], the lysosomal-endosomal system [37], or disorders of the immune system leading to chronic inflammatory processes including autoimmune processes [38,39] have all been considered to be causal candidates, but none have been demonstrated so far. The role of epigenetics, especially defects in histone homeostasis (acetylation and deacetylation) suggested by transcriptional dysregulation indicating changes in chromatin structure, which is seen in both murine ALS models and patients, has also been assumed [40]. In addition, some ALS patients have higher physical exertion and lower body mass indexes (BMI) compared to the unaffected population, which could be related to the onset [38,41].

In the case of FALS, an autosomal dominant pattern of inheritance with the most common mutation being in *C9orf72*, superoxide dismutase 1 gene (*SOD1*), and fused of the sarcoma (*FUS*) gene are usually seen [42]. Moreover, ALS has been shown to be associated with mutations in genes with DNA/RNA regulating functions, such as TAR DNA binding protein (*TARDBP*) [43,44].

Recently published studies reported selective hypothalamic atrophy in both SALS and FALS cases, including the asymptomatic stage of FALS [45]. In FALS, atrophy correlates with body mass index (BMI), but no correlation with the severity of motor problems has been found [45].

Early motor manifestations of SALS, with the presence of TDP-43 insoluble and ubiquitinated inclusions, reflect the failure of complex adaptive motor skills leading to split hand presentation, gait disorders, split leg syndrome, and bulbar symptomatology associated with vocalization [46].

A characteristic histopathological feature of TDP-43 pathology is its limitation to the cortical region and subcortical nuclei with direct cortical projections [47]. The pathological TDP-43 protein is found in the cerebral cortex, corticofugal fibers, subcortical nuclei, and the motor neurons of the brainstem and anterior horns of the spinal cord [39]. The prion-like mechanism at the synaptic terminals of corticofugal axons is currently accepted, and theoretically explains the spread to neocortical regions and the relationship between ALS and FTD [39].

Very rarely, paraneoplastic syndrome, positive for anti-Hu antibodies, and presenting as an MND, is found. MND is considered an atypical paraneoplastic syndrome that has yet to meet the criteria set by Graus et al., [48] i.e., at least one of three conditions have to be fulfilled: (1) Post-therapeutic reduction of neurological manifestations in the absence of immune modulation; (2) the presence of onconeural antibodies; and/or (3) partially characterized onconeural antibodies and a malignancy presenting within five years of the onset of neurological abnormalities [48].

## 4. Clinical Manifestation

Amyotrophic lateral sclerosis is an MND [49], and the typical form is characterized by upper and lower motoneuron involvement, which is present in 65–70% of cases. Another form is progressive bulbar paralysis with bulbar muscle involvement, which occurs in 25% of patients [50]. A rarer manifestation of the disease is progressive muscle atrophy with only lower motoneuron lesions; this occurs in 5–8% of ALS patients [39]. An extremely rare form of the disease is primary lateral sclerosis (PLS), characterized by a lesion limited to upper motoneurons and affecting 1–4% of patients [51]. In PLS, the clinical diagnosis is based on per exclusionem after excluding all other possible causes (structural, infectious, and demyelinating diseases leading to upper motor neuron syndrome or hereditary spastic paraplegias) [51]. Equally rare is the monomelic spinal amyotrophy variant with focal atrophy that usually presents as weakness in one limb [52,53].

Surprisingly often, i.e., in 55% of cases with clinically obvious dementia, 15% have been described [54] with characteristics of both ALS and cognitive impairment. In the dementia form (i.e., ALS-FTSD), five forms have been distinguished based on clinical presentation:

(1) ALS with cognitive impairment (ALSci);
(2) ALS with behavioral impairment (ALSbi);
(3) ALS with combined cognitive and behavioral impairment (ALS-cbi);
(4) Fully developed behavioral variant of frontotemporal dementia (bvFTD) in combination with ALS (ALS-FTD);
(5) Comorbid ALS and Alzheimer’s disease (AD) [55].

In the fully developed disease, the clinical picture is relatively characteristic, but diagnostic difficulties may occur at the beginning of the clinical manifestation. Objective findings in the developed form are a mixed picture of central and peripheral quadriparesis, bulbar symptoms, hyperreflexia, spasticity, positive pyramidal signs, atrophy of the muscles of the upper and lower limbs and tongue, and massive fasciculations, especially of the limbs and tongue [29].

The disease most often (in two-thirds of cases) [29] begins with an asymmetric weakness and wasting of limited muscle groups, with the subsequent development of spasticity [29] typically manifesting in the upper limbs [26]. Clinical findings may imitate mononeuropathy or radiculopathy; however, weight loss, fasciculations, emotional lability, and frontal-lobe cognitive impairment [56] may also be present. Bulbar onset with articulation disorders and swallowing difficulties [26] are seen in 30% of cases; additionally, striking atrophy and fasciculations of the tongue [57] may be present. Limb weakness may begin to develop simultaneously with bulbar symptomatology or with a delay of 1–2 years [29]. As paralysis progresses, it leads to death due to respiratory failure within 2–3 years in bulbar onset and 3–5 years in the more typical limb onset variant [29].

Less often, and in cases with a focal onset, muscle weakness affects the neck muscles [26], and muscle fatigue is a common symptom [58]. Fasciculations or cramps appear in the plexus muscles of the upper and lower limbs (e.g., deltoid muscle and quadriceps femoris muscle) [39]. Atrophies of small muscles of the hand and foot (especially the interosseous muscles) leading to split-hand or split-leg signs may be clinically present [59,60]. An ALS diagnosis requires the absence of sensory signs, visual disturbances, and sphincter problems [52].

In ALS-FTSD, the degree of cognitive impairment and behavioral manifestation varies from case to case and ranges from mild cognitive deterioration bordering on a normal finding to marked changes in behavior and personality with a severe frontal syndrome. Cognitive impairment is present mainly in the bulbar form of ALS [61].

Patients with ALS may develop some form of bvFTD during the disease; conversely, some patients with bvFTD develop symptoms of motor neuron disease (from clinically insignificant fasciculations with hyperreflexia to typical ALS). The onset of motor problems and dementia usually differ, making the clinical picture of simultaneous development of ALS and bvFTD unusual [62]. ALS-FTSD has a significantly worse prognosis and shorter survival than patients with isolated forms (i.e., ALS or bvFTD independently) [63].

## 5. Differential Diagnosis

To standardize the diagnosis for clinical research and reduce misdiagnoses, the El Escorial criteria have been implemented [64]. Regarding diseases belonging to an ALS differential diagnosis and brain-affecting diseases, adult polyglucosan body disease (APBD) has clinical symptomatology consistent with upper and lower motor neuron lesions. However, cognitive decline, distal sensory loss, and bladder and bowel function disturbances also occur, which can clinically help differentiate the entities [52].

Among brainstem and spinal cord diseases, ALS can be mimicked by multiple sclerosis, which can cause both upper and lower motoneuron symptoms, and in some cases, may even resemble bulbar onset ALS [52]. In the predominantly spinal form, an MRI of the cervical spinal cord is performed to exclude other possibly treatable conditions; however, cervical spondylotic myelopathy or syringomyelia may include dissociated sensory loss, which is usually present in syringomyelia but starts at a younger age than most ALS cases [65]. Another disease that should be considered in the differential diagnosis is degenerative myeloradiculopathy, which can also be consistent with the clinical presentation of ALS [52].

Spinal and bulbar muscular atrophy (SBMA), also known as Kennedy’s disease, is a hereditary X-linked lower motor neuron disease characterized by progressive muscular weakness and should also be considered in the differential diagnosis. An initial clinical manifestation usually includes muscle cramps, muscle twitching, tremor, fatigue, and slurred speech [66].

The potential for paraneoplastic encephalomyelitis, which can sometimes manifest as a motor neuron disorder, must not be overlooked. Sensory or autonomic features and ataxia may appear later in the course [67].

In dysphagia and dysarthria, which are neuromuscular transmission disorders (especially myasthenia gravis), arterial atherosclerotic disease, infiltrative tumors, and infectious or autoimmune causes should be ruled out [68]. Oculopharyngeal muscular dystrophy may also imitate ALS, although it usually involves the extraocular muscles and muscles of eyelids in contrast to ALS. In patients presenting with bulbar symptomatology lacking extraocular involvement, a muscle biopsy may be required [52].

Benign monomelic amyotrophy is a differential diagnosis for monomelic onset of ALS [52]. Generally, it is always necessary to examine the cerebrospinal fluid and perform an MRI of the brain and spinal cord. In addition to the rare variant of oculopharyngeal muscular dystrophy mentioned above, a muscle biopsy may be indicated to rule out polymyositis [69] or inclusion body myositis [70].

Furthermore, some systemic diseases can partially mimic the clinical manifestation of ALS—for example, hyperthyroidism, which can cause hyperreflexia as a corticospinal tract sign, fasciculations, weight loss, or weakness. However, many symptoms that are not typical for ALS are often present (fine tremor, tachycardia, heat intolerance, and anxiety). Weakness may also be seen in hyperparathyroidism and mimic the lower motoneuron onset form of ALS [52].

## 6. Types of ALS

### 6.1. Sporadic ALS

Sporadic ALS (SALS) is the most common form of ALS (90%)—it occurs randomly, without any known cause, with no clearly associated risk factors, and no family history of the disease, thus the etiology of most cases of SALS remains elusive.

#### 6.1.1. Background of SALS

Only 10% of SALS cases include known disease-associated ALS mutations [71], with *C9orf72* expanded alleles being the most common [24]. Mutations in the *SOD1* gene (gene for the antioxidant enzyme protecting cells from reactive superoxide radicals, endoplasmic reticulum stress, mitochondrial dysfunction, and axonal transport disruption) [72] are found in 2–3% of SALS [73]. Another interesting gene to study appears to be *TARDBP*, an abnormal TDP-43 fragment found in neurons and astrocytes in approximately 95% of SALS cases [74]. In less than 1% of SALS cases, mutations in hnRNP A1 and hnRNP A2B1 are detected. Oligogenic or polygenic SALS cases have an earlier age of onset [75]. The remaining cases likely represent the interplay of genetic and environmental factors.

#### 6.1.2. Gross Findings in SALS

**Figure 1:**
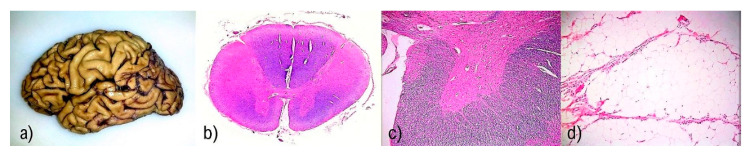
(**a**) Atrophy of the precentral gyrus together with atrophy of the frontal and temporal lobes may be present mainly in cases of ALS accompanied by dementia. (**b**) Marked atrophy and reactive astrogliosis of anterior horns, with atrophy of the anterior roots are the most striking findings in the standard staining method. Histochemical detection of myelin shows the most evident changes in the anterolateral cords in the forms of sclerosis. Stain: Luxol fast blue stain. (**c**) Detail of the described changes in anterior roots and horn. Stain: Luxol fast blue stain. The original magnification 200×. (**d**) Pronounced neurogenic atrophy and fatty pseudohypertrophy in the striated muscles of the respiratory muscles is a typical finding in ALS. Stain: Hematoxylin and eosin stain. The original magnification 200×.

SALS gross findings include typically shrunken ventral/anterior roots that appear grey compared to the dorsal/posterior roots, lateral corticospinal tract, and sometimes the whole spinal cord may appear atrophic [76] (see Figure 1).

The brain usually appears grossly normal, although there may be atrophy of the precentral gyrus. Atrophy is also evident on MRIs (on susceptibility-weighted images generated from gradient-echo pulse sequences (GRE/SWI)) as a bilateral hypointensity in the precentral gyrus, known as the “motor band sign [77].” In addition, atrophy of the frontal and temporal lobes may be seen, especially in cases clinically accompanied by dementia [78]. Brainstem atrophy, with a volume reduction of the medulla oblongata, may be noted as pontine atrophy. With neuroimaging methods, density reductions in the mesencephalic crura are apparent [79]. Moreover, severe atrophy of the muscles of the upper and lower limbs and tongue are also evident. All ALS variants share these gross findings; differences are described below in the relevant paragraphs.

#### 6.1.3. Neuropathological Findings in SALS

Degenerative changes are mostly observable in the motor area and by the large number of atrophied α-motor neurons located in the anterior grey matter of the spinal cord and the motor neurons of the cranial nerve nuclei in the brainstem [80]; these are striking histopathological signs of the disease, together with affected Betz cells in the primary motor cortex.

The loss of motor neurons leads to chronic denervation with neurogenic atrophy and fatty pseudohypertrophy in the striated muscles of the limbs and respiratory muscles; type 2 muscle fibers are the most vulnerable (Figure 1) [81,82]. Differentiation of muscle fibers is easy using adenosine triphosphatase (ATPase) staining, in which type 1 fibers appear lightly stained, and type 2 fibers appear darkly stained—this pattern is seen at pH 9,4 but not at pH 4,3 or 4,6 where the staining is inverse (type 1 fibers are dark, while type 2 fibers are pale) [83]. Assemblages of muscle fibers associated with re-innervation via axonal sprouting from neighboring motor axons are also typical; nevertheless, this compensatory mechanism is insufficient for muscle regeneration [84]. The location of atrophy is important; in the bulbar form, atrophy of the tongue, diaphragm, and intercostal muscles predominate, while in the typical form of ALS, atrophy in the limb muscles prevails. However, some, especially larger muscle fibers, can be paradoxically hypertrophic. In the late stages, clusters of pyknotic nuclei may be seen.

Standard staining is dominated by changes in the anterior horns, with marked atrophy, reactive astrogliosis, and atrophy of the anterior horns. In specialized histochemical detection of myelin, the most evident changes are seen in the anterolateral cords in the form of sclerosis; however, these changes can also be found in the posterior cords in longer disease courses [39]. A key diagnostic feature is the large number of atrophied large motor neurons in the anterior horns of the spinal cord, which is apparent in cervical and lumbar intumescence. The remaining neurons show signs of regressive changes, i.e., a tendency to wrinkle, the presence of lipofuscin deposits in the cytoplasm, and central chromatolysis in the nuclei [85]. A relatively characteristic feature is also phosphorylated neurofilament aggregates found as markedly swollen axons in anterior horns, generally referred to as spheroids [86].

Different types of inclusions can be found in the cytoplasm of affected neurons. Before immunohistochemical methods were routine, Bunina bodies, small eosinophilic granular inclusions, were considered diagnostically specific for ALS [87]. Now, the availability of immunohistochemical methods has broadened neuropathological examination. Anti-ubiquitin or anti-p62 [88] antibodies have revealed other types of inclusions widely present in motor neurons of the anterior spinal horns, in the brainstem, primary motor cortex, and neuronal structures in the frontal and temporal cortical areas [89]. The inclusions may be “skein-like,” [90] having an elongated or fibrous shape, which form bizarre spherical structures in the perikaryon of neurons, or light eosinophilic round hyaline inclusions. The presence of ubiquitin indicates impaired proteasomal degradation (in addition to RNA metabolism disorders at multiple levels) [91] as a key mechanism [92]. Most of the affected neurons and glial cells contain cytoplasmic TDP-43-immunoreactive inclusions [93]. The physiological TDP-43 protein acts as a DNA/RNA binding protein that binds both mRNA and DNA and mediates mRNA splicing, transcription, and translation [94]. Skein-like inclusions in anterior horn neurons and their neurites in the spinal cord may also be optineurin (OPTN) immunoreactive in both SALS and non-*SOD1* FALS [95].

Oligodendrocyte dysfunction is considered by some authors to be a primary contributing factor to ALS [96] as there is prominent degeneration of oligodendrocytes in the gray matter of the spinal cord in ALS-model mice before disease onset [97]. SOD1-dependent loss of oligodendrocytes, leading to the death of motoneurons, is discussed—firstly, it is considered to happen due to defective lactate release, and secondly, by influencing cell-to-cell contact between axons and enwrapping oligodendrocytes. When SOD1 is selectively removed from oligodendrocytes, delayed disease onset as well as a longer survival period in ALS-model mice is seen. This observation could also contribute to the theory about the relationship between the impaired function of oligodendrocytes and the vulnerability of motoneurons [97].

Considering pTDP-43, Brettschneider et al. recognized and described four stages of ALS based on the specific sequential pattern of pTDP-43. Stage I is characterized by lesions in the agranular motor cortex, brainstem motor nuclei of cranial nerves XII-X, VII, V, and spinal cord α-motoneurons. For stage 2, the involvement of the prefrontal neocortex (middle frontal gyrus), brainstem reticular formation, precerebellar nuclei, and the red nucleus is characteristic. Stage 3 shows pTDP-43 pathology in the prefrontal (gyrus rectus and orbital gyri) and postcentral neocortex plus striatum. The last stage is characterized by inclusions in the anteromedial part of the temporal lobe, including the hippocampus. At all stages, oligodendroglial aggregates are also present [98].

All forms of ALS share the neuropathological signs described above. Differences between the familial form and the form combined with dementia will be described in the following paragraphs (for summary see Table 1, Table 2 and Table 3).

### 6.2. Familial ALS

Familial ALS (FALS) is estimated to be 10% of all ALS cases [27], with the most common being autosomal dominant inheritance; however, as mentioned above, cases with autosomal recessive or X-linked inheritance have been described [23].

#### 6.2.1. Background of FALS

To date, 16 genes associated with FALS have been identified, and up to 80 different mutations have been detected in 10–20% of familial cases. Currently, the most important genes are associated with the metabolism of the TDP-43 protein (mostly hyperphosphorylated), which plays a crucial role in the pathogenesis of the disease. Although TDP-43 is a nuclear protein, it is transferred to the cellular cytoplasm after acute neuronal injury, where it forms stress granules [99]. In addition to the *TARDBP* gene itself, the fused-in-sarcoma (*FUS*) gene is involved in physiologically similar processes as *TARDBP* (i.e., a DNA/RNA binding protein involved in brain mRNA processing and transcription) and also plays a role in the pathogenesis of ALS [100]. Mutations in *SOD1* (the first gene historically identified as an ALS causing gene) [27], ALS2 (*ALSIN*; connected to juvenile-onset ALS [101], which is ALS starting before 25 years of age) [102], and senataxin (*SETX*; also seen in the juvenile form of ALS) are also associated with FALS [23]. Considering the juvenile variants, spatacsin (*SPG*) is typically associated with autosomal recessive heritability [23]. Optineurin (*OPTN*) is another important gene with autosomal recessive heritability [23]. Moreover, vesicle-associated membrane protein B (*VAPB*), valosin-containing peptide (*VCP*), progranulin (*PGRN*), ubiquilin 2 (*UBQLN2*; a mutation leading to ubiquitin-mediated disruption of proteasomal degradation) [103], and sequestosome 1 (*SQSTM1/p62* [104]; involved in the degradation of misfolded proteins through ubiquitin–proteasomes [105] or the autophagy-lysosome system) [106,107] are also seen in FALS. The relatively recently discovered the 72nd open reading frame on chromosome 9 (*C9orf72*) [108] has now been found in many patients with FALS, with the incidence estimated to be up to 20%. Patients with the *SOD1* mutation rarely suffer from cognitive impairment [109].

#### 6.2.2. Neuropathological Findings in FALS

In addition to the above-mentioned neuropathological features shared with SALS, some cases of FALS have hyaline inclusions similar to Lewy bodies in the cytoplasm of neurons. They are strongly immunoreactive with anti-superoxide dismutase (SOD1) antibodies in those cases with the *SOD1* gene mutation. Inclusions immunoreactive to SOD1 are located in lower motor neurons and represent a striking pathological feature of FALS; they are due to *SOD1* mutations and are also seen in *SOD1* murine models [110]. Neuropathologically, FALS caused by the *SOD1* mutation can be distinguished from the sporadic disease by the relative sparing of the motor cortex, slight to mild corticospinal tract involvement, which is in contrast with severe atrophy of the anterior roots, and the degeneration of lower motor neurons in the sporadic form of the disease [27].

**Figure 2:**
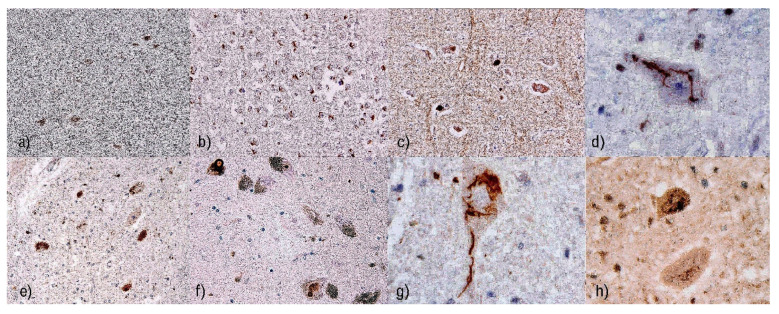
(**a**) Overview of inclusions positive in reaction with anti-phosphorylated TDP-43 (pTDP-43) antibody, original magnification 100×. (**b**) pTPD-43 positive cytoplasmic inclusions in the hippocampus are considered one of the characteristic features of the variant with *C9orf72* mutation, magnification 100×. (**c**) Cytoplasmic pTDP-43-positive cytoplasmic inclusions in the hypoglossal motor neurons, original magnification 200×. (**d**) Detailed shape of cytoplasmatic pTDP-43-positive inclusion in the lower motor neuron. The original magnification 400×. (**e**) Inclusions detected with anti-p62 antibodies in the mesencephalic nuclei. The original magnification was 100×. (**f**) Inclusions detected by anti-p62 antibody seen in the neurons of mesencephalic nuclei. The original magnification 200×. (**g**) Detailed morphology of p62-positive inclusions in the cytoplasma of upper motor neuron, original magnification 400×. (**h**) Morphology of ubiquitin-positive “skein-like” inclusions with characteristic elongated or fibrous shape, which form bizarre spherical structures in the perikaryon of neurons. The original magnification was 400×.

TDP-43 cytoplasmic inclusions, mainly found in the pyramidal, frontal, and temporal cortex, and hippocampal areas, are considered characteristic features of FALS with the *C9orf72* mutation; the cerebellar granule cell layer, hippocampal pyramidal neurons, and neocortex all contain ubiquilin, p62, and ubiquitin inclusions that are negative relative to the TDP-43 reaction (Figure 2) [93].

Ubiquilin-positive (ubiquitin-like-positive) inclusions have been identified in spinal cord sections of patients with X-linked FALS; the inclusions co-localize with ubiquitin itself, p62, TDP-43, FUS, and OPTN but not with SOD1. UBQLN2 immunoreactivity has also been observed in spinal cord sections of sporadic ALS, ALS with dementia, and non- *SOD1* FALS.

### 6.3. Amyotrophic Lateral Sclerosis-Frontotemporal Spectrum Disorder

In patients with amyotrophic lateral sclerosis-frontotemporal spectrum disorder (ALS-FTSD), inclusions positive for the anti-TDP-43 antibody reaction are usually present, so the clinical and neuropathological designation of FTLD-MND-TDP (frontotemporal lobar degeneration with motor neuron disease and TDP-43 positive inclusions) is used. However, dementia syndrome is defined as a loss of cognitive functioning and behavioral abilities to such an extent that it interferes with daily life and activities. The behavioral form (ALSbi) does not meet the condition of deterioration of self-sufficiency. Moreover, cognitive decline is often associated with a poorer prognosis [111]. Recently, the terminology was changed from “FTLD-MND” to “ALS-FTSD.” At present, the term FTLD-MND-TDP is purely neuropathological, while in clinical practice, ALS-FTSD is used [21].

#### 6.3.1. Background of ALS-FTSD

**Figure 3:**
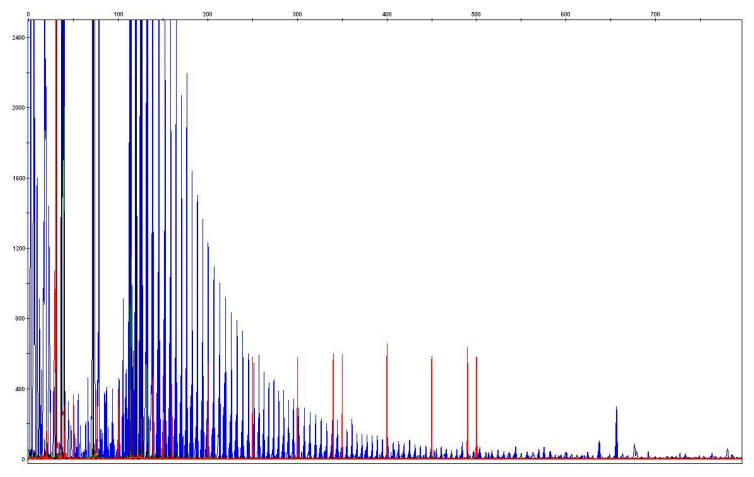
Determination of copy number of hexanucleotide expansion of C9orf72. Reference values: Normal allele <20 GGGGCC repeats, permuted allele 20–100 GGGGCC repeats, complete penetrance> 100 GGGGCC repeats. The blue arrow indicates the location of 20 GGGGCC repeats and the red arrow indicates the location of 30 or more repeats.

A strong genetic burden is evident in ALS-FTSD cases [112]. The pathological expansion of the “hexa-repeat” (GGGGCC) in chromosome 9 open reading frame 72 is closely related to the behavioral variant of frontotemporal dementia (bvFTD), which occurs either in direct association with ALS or as a purely cognitive impairment lacking motor neuron dysfunction. Carriers of *C9orf72* mutations have a significantly higher risk of developing cognitive impairment (40–50%) than patients lacking this mutation (8–9%) [113,114]. The spectrum of involvement in families carrying *C9orf72* gene expansions (Figure 3) differs significantly from typical ALS based on purely behavioral symptomatology (bvFTD), i.e., without demonstrable motor neuron involvement up to a combination of ALS and cognitive impairment [115]. In patients with ALS with psychosis and anosognosia, the presence of *C9orf72* mutation should be considered [21,116].

#### 6.3.2. Neuropathological Findings in ALS-FTSD

We can distinguish three basic types of FTLD depending on the hallmark pathological protein: (1) FTLD-tau (characterized by tau-positive inclusions), (2) FTLD-TDP (with TDP-43 inclusions), and (3) FTLD-FUS (having FUS-positive inclusions); although, a small proportion of FTD cases do not express any of the above-mentioned proteins. Those that react with ubiquitin or other markers of the ubiquitin-proteasome system are called FTLD-UPS, while completely immuno-negative cases are grouped as FTLD-NOS (not otherwise specified) [117]. Widespread ubiquilin-positive inclusions are also usually observed in the hippocampal region, including patients with the *C9orf72* mutation [103].

Most ALS cases belong to the FTLD-TDP group and exhibit TDP-43 immunoreactive inclusions [118,119]; the remaining cases are in the FTLD-FUS group.

The Strong criteria [63] for the neuropathological diagnosis of ALS-FTSD remain unchanged, including the examination of the brain and spinal cord; AD must always be considered. A p62 and dipeptide repeat (DPR) pathology in the cerebellum and hippocampus is pathognomonic for *C9orf72*-linked ALS-FTSD [120]. The other neuropathological findings are identical to those cases lacking cognitive impairment [121]. It is probably not surprising that in genetic ALS-FTSD cases, SOD1- or FUS-immunoreactive inclusions can be found; moreover, alterations in microtubule-associated tau protein (tau) metabolism have been observed in ALS-FTSD, with the presence of tau deposits usually in the hyperphosphorylated form [92]. Since the primary function of tau is to provide stability to microtubules, site-specific tau phosphorylation therefore affects the interaction of tau and microtubules. Pathological hyperphosphorylation reduces the number of interactions between tau and microtubules, allowing tau to form less soluble oligomers and subsequently leading to the formation of fibrils [122]. In addition, research on murine models shows that having coexisting pathologies in TDP-43 and tau metabolism potentiate each other [92]. Thus, it is clear that FTD and ALS can share clinical manifestations and underlying pathophysiology, which are reflected in changes in TDP-43 and tau metabolism.

In FTLD-TDP-MND, inclusions are most common in the frontal and temporal cortex. In ALS with preserved cognition, deposits are located predominantly in the cytoplasm of motor neurons without affecting cortical structures [121].

According to the current neuropathological systemic classification, a harmonized classification system recognizes four types of FTLD-TDP pathology. Type A corresponds to Mackenzie et al. type 1 and Sampathu et al. type 3 and contains numerous short dystrophic neurites (DNs) and oval or crescent neuronal cytoplasmic inclusions (NCIs), which are located primarily in the second neocortical layer. Moderate numbers of lentiform neuronal intranuclear inclusions (NIIs) can also be present, although they are not a consistent sign of this subtype. The clinical phenotype of type A is bvFTD or progressive non-fluent aphasia and is based on mutations in the progranulin gene.

For this article, type B, equivalent to Mackenzie et al. type 3 and Sampathu et al. type 2, is the most important. Type B is characterized by moderate numbers of NCI, distributed throughout all cortical layers, with very few DNs. This condition is associated with a genetic defect in the short arm of chromosome 9, and clinically manifests as bvFTD or MND with FTD.

Type C is equivalent to Mackenzie et al. type 2 and Sampathu et al. type 1; it has a predominance of elongated DNs in the upper cortical layers and few NCIs; it leads to bvFTD or semantic dementia.

Type D is associated with the inclusion of body myopathy of Paget’s disease of the bone and frontotemporal dementia caused by *VCP* mutations; there are large numbers of short DNs and numerous lentiform NIIs.

However, there are other examples of the wide-range neuropathological background of ALS/MND clinical symptomatology. The most important neurodegenerations in this field are tauopathies. Recently, we described tau protein deposits in corticospinal tract structures in patients with progressive supranuclear palsy (PSP) clinically mimicking MND. [123] Moreover, case reports of globular glial tauopathy (GGT) with the presence of tau-positive globular oligodendroglial inclusions (GOIs) with partial overlapping neuropathological features of progressive supranuclear palsy (PSP), clinically manifesting as MND and/or FTD, exist [124]. Neuronal, astrocytic, and especially oligodendroglial 4-repeat (4R) tau positive, mostly Gallyas negative, inclusions are present, whereas the GOIs in white matter correlate with the severity of neuropathologically confirmed degeneration [124]. Massive oligodendroglial involvement leading to changes in white matter (pallor, axonal loss, and gliosis) implies that these cases may represent primary oligodendrogliopathy [124]. Some authors also speculate that this entity could be an equivalent of “tau” multiple-system atrophy [125].

Considering comorbid cases and dementia in ALS, some comorbid ALS/AD cases have appeared in the literature; for example, one study reported that about 50% of ALS cases had Aβ deposits, and at least half of the cases had neuritic plaques in the neocortex [126]. Those patients with comorbid ALS/AD usually have progressive amnestic dementia, which is typical for AD and is accompanied by early distinctive impairment of episodic memory; MRIs of the hippocampus show significant atrophy [55].

Comorbid ALS/dementia with Lewy bodies (DLB) also exists. The prevalence of the DLB pathology in ALS is higher than in the general population, and it has been reported that Parkinsonian features develop in about 30% of ALS patients [127]. Levodopa-responsive parkinsonism accompanied by ALS, with symptoms that appear later in the course of the disease, is known as Brait–Fahn–Schwarz disease [128]. The incidence of this syndrome is high on the Japanese Kii Peninsula and the Micronesian island of Guam but is rare elsewhere in the world [129], although, Brait–Fahn–Schwarz syndrome was diagnosed in the Czech Republic (personal experience, not published yet).

## 7. Molecular Biomarkers of ALS

It seems that CFS-PGRN levels can be used to predict the type of FTD as it mirrors the FTD-subtype—the level is significantly lower in cases with TDP-43 pathology lacking a *GRN* mutation [130]. CSF-TDP-43 is also considered to be a promising CSF biomarker for ALS-FTD cases [131]. Interestingly, some researchers report higher CSF-TDP-43 levels in ALS cases than in FTLD, which might indicate more faster progression of TDP-43 pathology in ALS [132].

To predict disease development, some of the above-mentioned molecules can be used as biomarkers. Mentioned markers from the group of RNA-binding proteins in ALS are TDP-43, FUS, or hnRNPs, although some others can be used—TATA-box binding protein associated factor 15 (TAF15), which is mutated in both SALS and FALS; Ewing Sarcoma breakpoint region 1/EWS RNA binding protein 1 (EWSR1) with similar characteristics as FUS and TAF15 that they can easily aggregate leading to the toxicityn [133]; or ataxin-2 (ATXN2), which acts as a dose-sensitive modifier of TDP-43 toxic effect [134]. From the group of ALS-related genes, we could highlight *SOD1*, *C9orf72*, or spastacsin (*SPG*) [133], as well as non-coding RNA such as microRNA, circular RNA [133], or other molecules. It is worth mentioning serum uric acid, whose serum levels negatively correlate with the risk of death in patients with ALS [135]; the detection of higher levels of chitotriosidase in CSF correlates with microglial activation in the white matter of the spinal cord and may be helpful in patients with a short history of symptoms that are difficult to identify [136]. The identification of the blood neurofilament light chain (NFL) positively correlates with the progression of the disease, and a shorter survival period is indicated by increased NFL [137]. Some studies also indicate that patients with ALS have significantly higher levels of CSF total tau protein and a lower phosphorylated tau/total tau ratio than the control population [138].

## 8. Conclusions

Although much about the pathogenesis of ALS remains elusive, some major molecular pathways that can trigger selective damage in specific neurons are already known. Moreover, the disruption of individual ALS-causing genes can lead to the formation of pathological inclusions. Recognition of these features can help determine final diagnoses and also indicate potential links with other neurodegenerative diseases that need to be simultaneously considered.

## Figures and Tables

**Table 1:** Summary of characteristics genes and inclusions in SALS cases.

Disease-Associated Genes	Inclusions
✓ mutations in 10% of cases ✓ SOD1 ✓ C9orf71 ✓ TARDBP ✓ hnRNP A1 + hnRNP A2B1 (< 1%)	✓ SOD1 ✓ TDP-43 ✓ ubiquitin ✓ p62 ✓ OPTN (in non-SOD1 cases)

**Table 2:** Summary of characteristics genes and inclusions in FALS cases.

Disease-Associated Genes	Inclusions
✓ mutations up to 20% ✓ most common—Autosomal dominant inheritance; however, cases with autosomal recessive or X-linked inheritance exist ✓ SOD1 (cognitive impairment rarely) ✓ C9orf72 ✓ TARDBP ✓ FUS ✓ UBQLN2 ✓ p62 ✓ ALSIN (juvenile-onset) ✓ SETX (juvenile onset) ✓ SPG (autosomal recessive) ✓ OPTN (autosomal recessive) ✓ VAPB ✓ VCP ✓ PGRN	✓ SOD1 ✓ FUS ✓ TDP-43 (different than ubiquitin, ubiquilin and p62 positive inclusions) ✓ ubiquitin ✓ p62 ✓ OPTN ✓ Ubiquilin—co-localize with ubiquitin itself, p62, TDP-43, FUS, and OPTN but not with SOD1

**Table 3:** Summary of characteristics genes and inclusions in ALS-FTSD cases.

Disease-Associated Genes	Inclusions
✓ C9orf72 (40–50% risk of development of cognitive impairment)	✓ tau ✓ FUS ✓ TDP-43 ✓ ubiquitin ✓ p62

## Data Availability

The authors confirm that all data underlying the findings are fully available without restriction. All data are included within the manuscript.
